# NGS-based targeted gene mutational profiles in Korean patients with pancreatic cancer

**DOI:** 10.1038/s41598-022-24732-2

**Published:** 2022-12-03

**Authors:** Kwangrok Jung, Sejoon Lee, Hee Young Na, Ji-Won Kim, Jong-Chan Lee, Jin-Hyeok Hwang, Jin Won Kim, Jaihwan Kim

**Affiliations:** 1 Department of Internal Medicine, Seoul National University Bundang Hospital, Seoul National University College of Medicine, 82, Gumi-Ro 173 Beon-GilGyeonggi-Do, Seongnam-Si, 13620 Republic of Korea; 2 Department of Pathology and Translational Medicine, Seoul National University Bundang Hospital, Seoul National University College of Medicine, Seongnam, Republic of Korea; 3 Precision Medicine Center, Seoul National University Bundang Hospital, Seongnam, Republic of Korea

**Keywords:** Cancer, Computational biology and bioinformatics, Gastroenterology, Oncology

## Abstract

According to molecular profiling studies, a considerable number of patients with pancreatic cancer harbor potentially actionable mutations. However, there are limited relevant data from the Korean population. We assessed the molecular profiles of patients with pancreatic cancer in Korea. This study collected molecular profiling data from patients with pancreatic cancer who visited Seoul National University Bundang Hospital between March 2018 and August 2020. Formalin-fixed, paraffin-embedded tumor specimens were sequenced using a targeted next-generation sequencing (NGS) platform. Cancer-associated mutations were analyzed, and potentially actionable mutations were identified. Potentially actionable mutations were classified into “highly actionable” and “modifies options” based on the Know Your Tumor registry study. In total, 87 patients with NGS tumor panel data were identified. Sixty-one patients (70.1%) had metastatic disease at the time of tissue acquisition. Tissues were obtained from the primary tumors and metastatic sites in 41 (47.1%) and 46 (52.9%) patients, respectively. At least one pathogenic mutation was reported in 86 patients (98.9%). The frequencies of four common mutations in our cohort were similar to those in The Cancer Genome Atlas data. Potentially actionable mutations were identified in 27 patients (31.0%). Of these, mutations categorized as highly actionable and modifies options were identified in 12 (13.8%) and 18 patients (20.7%), respectively. The most frequent highly actionable mutations were located in DNA damage response genes, such as *BRCA1*, *BRCA2*, or *ATM* (*n* = 6, 6.9%). Two patients with germline *BRCA1* mutations received maintenance poly(adenosine diphosphate-ribose) polymerase inhibitor therapy. One patient has been receiving maintenance treatment for 18 months while remaining in radiologically complete remission. Mutational profiles using targeted NGS in Korean patients with pancreatic cancer were similar to those in Western patients. The present study supports the clinical potential and possible expanded clinical use of genetic profiling.

## Introduction

Pancreatic cancer is a lethal cancer with a 5-year survival rate of only 10% in the United States^1^. The incidence and death rates of pancreatic cancer are gradually increasing, and it is expected to become the second leading cause of cancer-related death in the United States by 2030^1,2^. In Korea, in 2019, pancreatic cancer was reported to be the eighth most common cancer with more than 8000 new cases per year^3^. Moreover, it was reported to be the fifth most common cause of cancer-related death, and the 5-year survival rate was only 13.9% in 2019. The main obstacle in the improvement of the poor prognosis of pancreatic cancer is that less than 20% of patients have resectable lesions at diagnosis, and the median overall survival of patients with advanced stage is less than 1 year with the current combination chemotherapy^1,4,5^.

Recent molecular profiling studies in pancreatic cancer revealed clinically relevant recurrent molecular alterations and genomic subtypes, which are collectively termed actionable mutations^6–11^. They reported that approximately 25% of patients with pancreatic cancer harbor potentially actionable mutations, and mutations were most commonly present in DNA damage response (DDR) genes. Additionally, the recently reported Know Your Tumor (KYT) registry trial, which compared survival between patients who received matched therapy based on molecular testing and those who received unmatched therapies, revealed improved survival outcomes with molecular profiling-based precision medicine in pancreatic cancer^12^.

Although several studies have described the potential and clinical usefulness of molecular profiling in pancreatic cancer, the data related to Asian populations remain limited. Recently, a clinical next-generation sequencing (NGS) platform was implemented in daily practice, and molecular profiling data in pancreatic cancer are accumulating. Therefore, we aimed to report the results of molecular profiling for pancreatic cancers at a single tertiary center in Korea as well as to share the relevant clinical experiences.

## Materials and methods

### Patients and tumor specimens

This retrospective cohort study included patients pathologically diagnosed with pancreatic cancer who underwent NGS-based targeted gene mutational assays between March 2018 and August 2020 at Seoul National University Bundang Hospital (SNUBH). Formalin-fixed, paraffin-embedded tumor specimens were obtained for NGS at the time of diagnosis or when the disease recurred or progressed. Biopsies were conducted for primary pancreatic tumors or metastatic lesions. Paired germline testing using blood samples was performed at the discretion of the attending physician.

### NGS panel information and data analysis

Tumor tissue specimens were sequenced on the SNUBH pan-cancer panel, a targeted sequencing platform at SNUBH. The first three patients were profiled on SNUBH ver. 1.1, which targeted 89 genes, and subsequent patients were profiled on SNUBH ver. 2.0, which targeted 544 genes (Supplementary Table S1). Microsatellite instability (MSI) and tumor mutational burden (TMB) were reported only in SNUBH ver. 2.0.

Samples were sequenced on the MiseqDx platform (Illumina, San Diego, CA, USA) for the SNUBH panel ver. 1.1 and the NextSeq 550Dx platform (Illumina) for the SNUBH panel ver. 2.0. Reads were aligned to the human reference genome hg19. Mutect2 was used to detect single nucleotide variants (SNVs) and small insertion/deletions (INDELs), and SnpEff was used to annotate the identified variants. Only SNVs/INDELs with variant allele frequencies of ≥ 2% were selected. CNVkit was used to identify copy number variation (CNV), and a mean copy number of ≥ 5 was considered gain (amplification). Gene fusions were identified using LUMPY, and read counts ≥ 3 were interpreted as positive results for the structural variations.

The MSI phenotype was detected using mSINGs, and TMB was calculated as the number of eligible variants in the effect panel size (1.41 megabases). Eligible variants were missense mutations with the following criteria: 1) variants reported in the population database with a frequency of > 1% (East Asian, gnomAD) were excluded; 2) pathogenic and likely pathogenic mutations reported in ClinVar were excluded; 3) variants with allele frequencies of less than 2% were excluded; and 4) variants with a depth of less than 200 were also excluded.

All genetic alterations were reported and classified using a tiered system according to the standardized guideline for the interpretation and reporting of sequence variants in cancer as follows: 1) Tier I, variants of strong clinical significance such as those targeted by FDA-approved, professional guideline, or well-powered study-reported therapies; 2) Tier II, variants of potential clinical significance such as those targeted by FDA-approved treatments for different tumor types or investigational therapies; 3) Tier III, variants of unknown clinical significance; and 4) Tier IV, benign or likely benign variants^13^.

### Potentially actionable mutations

All genetic alterations were reviewed, and potentially actionable mutations were analyzed. The gene list for potentially actionable mutations was constructed according to the previous KYT registry study (Supplementary Table S2)^9^. Mutations were classified as “highly actionable” or “modifies options” as described in the KYT study. Specifically, mutations with clinical evidence of a high response rate in patients with relevant molecular abnormalities in any cancer type were considered highly actionable, and those possibly implicated in the response to therapy based on the underlying mechanism were classified as modifies options. In addition, patients with MSI-high status or high TMB (which was defined as > 20 mutations per megabase) were examined.

### Statistical analysis

For the comparison of mutation frequency according to datasets or clinical settings, the chi-square or Fisher’s exact test was used, and Bonferroni’s correction was applied for multiple comparisons. All statistical analyses and mutational mapping in this study were performed using the open software R version 4.0.3 (R Foundation for Statistical Computing, Vienna, Austria).

### Ethical approval

The Institutional Review Board of SNUBH approved this study (IRB no. B-2007–622-108) and waived the requirement for written informed consent from the participants because of the retrospective nature of this study. This study was conducted in accordance with the principles of the Declaration of Helsinki, and all study procedures were conducted following the relevant guidelines and regulations.

## Results

### Baseline characteristics of the patients

Between March 2018 and August 2020, 87 patients were enrolled in this study. Patient characteristics and tumor specimen information are summarized in Table 1. The median age (range) of patients at diagnosis was 64 (35–86) years, and 54 patients (62.1%) were men. Meanwhile, 61 (70.1%) and 26 (29.9%) patients had metastatic and non-metastatic pancreatic cancers at the time of tissue acquisition, respectively. Adenocarcinoma was the dominant type of cancer (*n* = 82, 94.3%).

**Table 1:** Patient characteristics and tumor specimen information.

	Total (*n* = 87)
Age (years), median	64 (35–86)
Male	54 (62.1)
**Stage at tissue acquisition**	
Non-metastatic	26 (29.9)
Metastatic	61 (70.1)
**Cell type**	
Adenocarcinoma	82 (94.3)
Adenosquamous carcinoma	4 (4.6)
Mucinous carcinoma	1 (1.1)
**Site of tissue acquisition**	
Primary tumor	41 (47.1)
Liver	33 (37.9)
Peritoneum	5 (5.7)
Others*	8 (9.2)
**Method of tissue acquisition**	
Ultrasound-guided needle biopsy	36 (41.4)
Surgical resection	25 (28.7)
Fine-needle aspiration/biopsy	20 (23.0)
Others**	6 (6.9)
Prior chemotherapy before tissue acquisition	13 (14.9)

Data are presented as the median (range) or number (%).

*Stomach (*n* = 2), duodenum (*n* = 2), neck lymph node (*n* = 2), bile duct (*n* = 1), and lungs (*n* = 1). **Biopsy from stomach or duodenum invasion (*n* = 4), biopsy from the bile duct through endoscopic retrograde cholangiopancreatography (*n* = 1), and percutaneous core needle biopsy of lung metastasis (*n* = 1).

Tumor specimens for NGS were obtained from primary tumors and metastases in 41 (47.1%) and 46 patients (52.9%), respectively. The liver was the most frequent metastatic site for tissue acquisition (*n* = 33, 37.9%). Concerning the method of tissue acquisition, ultrasound-guided percutaneous needle biopsy (*n* = 36, 41.4%) was most common, followed by surgical resection (*n* = 25, 28.7%) and fine-needle aspiration or biopsy (*n* = 20, 23.0%). Meanwhile, 13 patients (14.9%) underwent additional biopsy for NGS after cancer progression or recurrence.

### Landscape of genetic mutations

**Figure 1:**
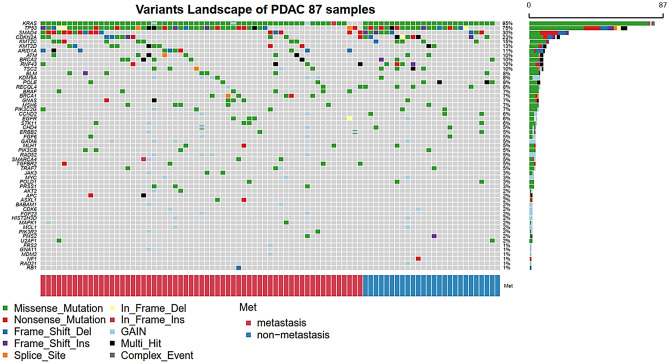
Landscape of genomic alterations in patients with pancreatic cancer (*n* = 87). Distribution of recurrent single nucleotides variants (SNVs)/insertions/deletions (INDELs) and copy number variation (CNV) as well as the total percentage of patients with SNVs/INDELs or CNV for each recurrent mutated gene.

The mean depth of coverage in targeted gene sequencing ranged from 259 to 1,717. The most frequently reported mutation was *KRAS* mutation, which was found in 82 patients (94.3%), followed by *TP53* mutations in 65 patients (74.7%), *SMAD4* mutations in 26 patients (29.9%), and *CDKN2A* mutations in 20 patients (23.0%) (Fig. 1). The frequencies of these four mutations were similar to those reported in The Cancer Genome Atlas (TCGA) dataset (Table 2)^14^. Additionally, the frequencies of other significant recurrent mutations (which were previously reported in the TCGA dataset), such as *RNF43* (10.3% vs. 6.0%), *ARID1A* (11.5% vs. 5.3%), *TGFBR2* (4.6% vs. 5.3%), and *GNAS* (6.9% vs. 6.7%) mutations, were not significantly different from those of the TCGA dataset. CNVs were identified in 21 patients (24.1%), and *KDM5A* amplification (*n* = 5, 5.7%) was the most common amplification, followed by *GATA6* (*n* = 4, 4.6%), *RAD52* (*n* = 4, 4.6%), *KRAS* (*n* = 3, 3.4%), and *MYC* amplifications (*n* = 3, 3.4%) (Fig. 1). The mutation frequency was compared according to various clinical characteristics, including cancer stage, biopsy site, method of tissue acquisition, and prior chemotherapy; however, no significant differences in the frequencies of any mutations were identified (Supplementary Tables S3A and S3B).

**Table 2:** Comparison of the frequency of common recurrent mutations with TCGA dataset.

Genes	TCGA (*n* = 150)	%	SNUBH (*n* = 87)	%	Adjusted *P* value
*KRAS*	136	90.7	82	94.3	0.999
*TP53*	104	69.3	65	74.7	0.999
*SMAD4*	37	24.7	26	29.9	0.999
*CDKN2A*	22	14.7	20	23.0	0.999
*GNAS*	10	6.7	6	6.9	0.999
*RNF43*	9	6.0	9	10.3	0.999
*ARID1A*	8	5.3	10	11.5	0.844
*TGFBR2*	8	5.3	4	4.6	0.999
*ATM*	5	3.3	9	10.3	0.273
*DNMT3A*	5	3.3	1	1.1	0.999

At least one Tier I or Tier II mutation was identified in 86 patients (98.9%). Other Tier I or II SNV/INDELs in oncogenes, such as *BRAF* (*n* = 1, 1.1%), *GNAS* (*n* = 1, 1.1%), and *EGFR* (*n* = 1, 1.1%), and tumor suppressor genes, such as *ARID1A* (*n* = 3, 3.4%) and *RNF43* (*n* = 2, 2.3%), were observed, except for the four common mutations. Most common Tier I or Tier II CNVs were *MYC* and *KRAS* amplifications, where were observed in three patients (3.4%) each, followed by *CDK6*, *CCND2*, *ERBB2, FGF6, FGF23,* and *MCL1* amplifications, which were observed in two patients (2.3%) each. Tier I or II mutations related to hereditary cancer syndrome in pancreatic cancer were identified in *BRCA1* (*n* = 2, 2.3%), *BRCA2* (*n* = 2, 2.3%), *PRSS1* (*n* = 1, 1.1%), *MSH6* (*n* = 2, 2.3%), *PMS2* (*n* = 1, 1.1%), and *APC* (*n* = 1, 1.1%). Among them, two patients with *BRCA1* mutations were confirmed to have germline pathogenic variants through paired germline testing using blood samples.

### Potentially actionable mutations

**Figure 2:**
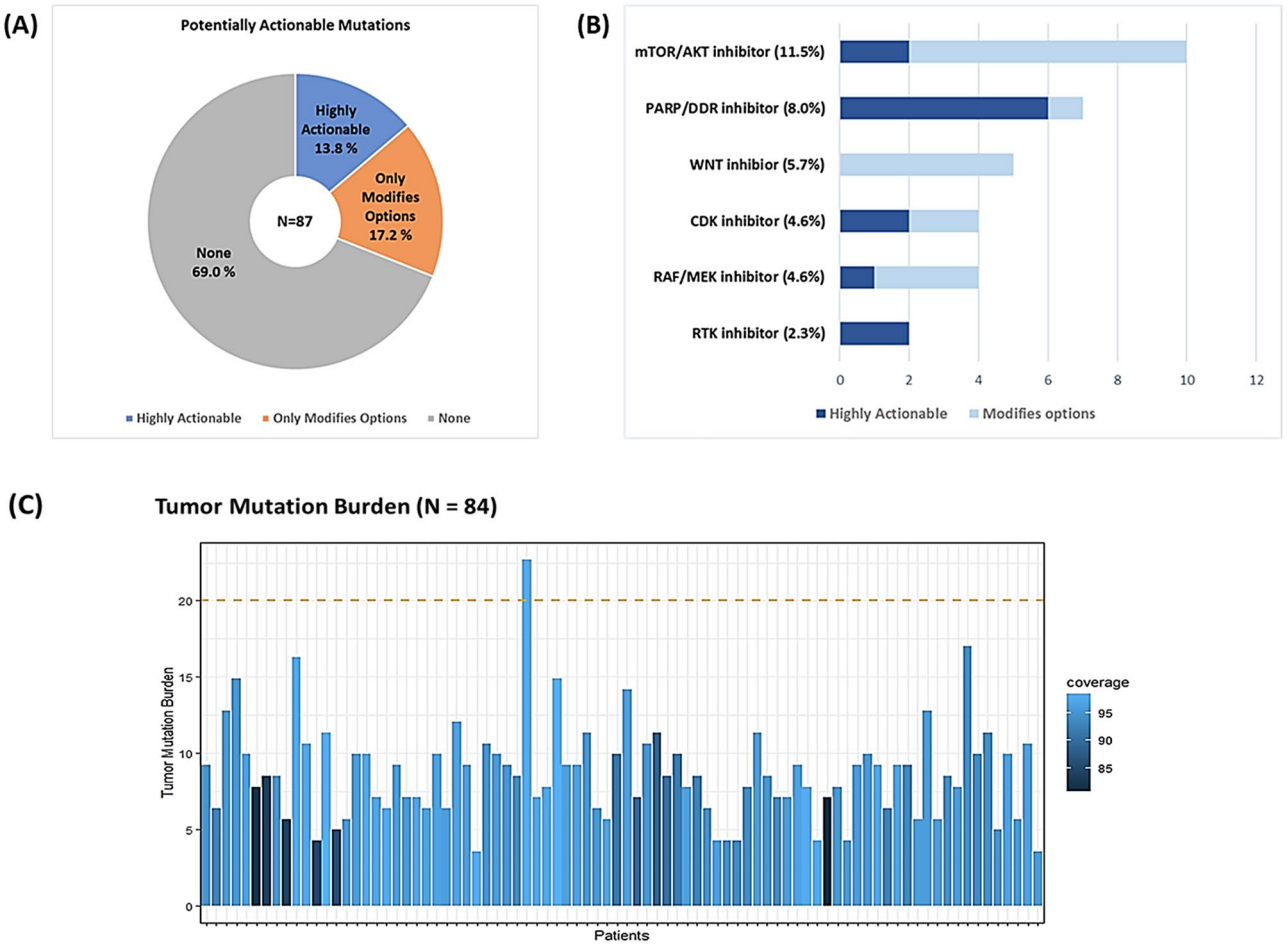
(**A**) The percentage of patients with potentially actionable mutations (*n* = 87). (**B**) Therapeutic options according to the mutational profile (*n* = 87). (**C**) Tumor mutation burden profiling (*n* = 84).

In total, 27 patients (31.0%) were identified to have at least one potentially actionable mutation (Fig. 2A and Supplementary Table S4). Of these, highly actionable and modifies options mutations were found in 12 (13.8%) and 18 patients (20.7%), respectively. After excluding three patients (3.4%) who had both highly actionable and modifies options mutations, there were 15 patients with only modifies options mutations (17.2%).

Highly actionable mutations included mutations in *BRCA1* (*n* = 2), *BRCA2* (*n* = 2), *ATM* (*n* = 2), and *BRAF* (*n* = 1) and amplification in *CDK6* (*n* = 2), *ERBB2* (*n* = 2), and *AKT2* (*n* = 1). Mutations in DDR genes such as *BRCA1*, *BRCA2*, and *ATM*, which were observed in six patients, were the most frequent highly actionable mutations. Concerning modifies options, mutations in *ARID1A* (*n* = 7) were the most frequent, followed by *RNF43* mutations (*n* = 5), *GNAS* mutations (*n* = 2), and *CCND2* amplification (*n* = 2). When classified by therapeutic options, mTOR/AKT inhibitors represented the most common drug class (*n* = 10, 11.5%), followed by poly(adenosine diphosphate-ribose) polymerase (PARP) inhibitors (*n* = 7, 8.0%), WNT inhibitors (*n* = 5, 5.7%), CDK inhibitors (*n* = 4, 4.6%), and MEK inhibitors (*n* = 4, 4.6%) (Fig. 2B). The MSI status and TMB were analyzed in 84 patients profiled with the SNUBH panel ver. 2.0. No patient had MSI-high tumors, but high TMB was reported in one patient (1.2%) (Fig. 2C).

### Clinical implications of NGS results

In this study, three patients (3.4%) received matched therapy based on their molecular profiles. Of these, one patient was a 65-year-old man who was diagnosed with metastatic pancreatic cancer with an *EGFR* exon 19 deletion. He received a combination treatment, including gemcitabine and an *EGFR* tyrosine kinase inhibitor erlotinib (Roche, Basel, Switzerland); however, he showed rapid aggravation just after one cycle of the treatment.

The remaining two patients were diagnosed with metastatic pancreatic cancer using percutaneous needle biopsy of liver metastasis, and targeted gene profiling with the SNUBH panel ver. 2.0 was conducted at the time of diagnosis. The profiling results illustrated that both patients had tumors with Tier I *BRCA1* mutations. With additional germline testing, they were confirmed to have germline pathogenic *BRCA1* mutations, and they received the PARP inhibitor olaparib (AstraZeneca, Cambridge, United Kingdom) as maintenance therapy after FOLFIRINOX chemotherapy, as introduced in the POLO trial^15^.

**Figure 3:**
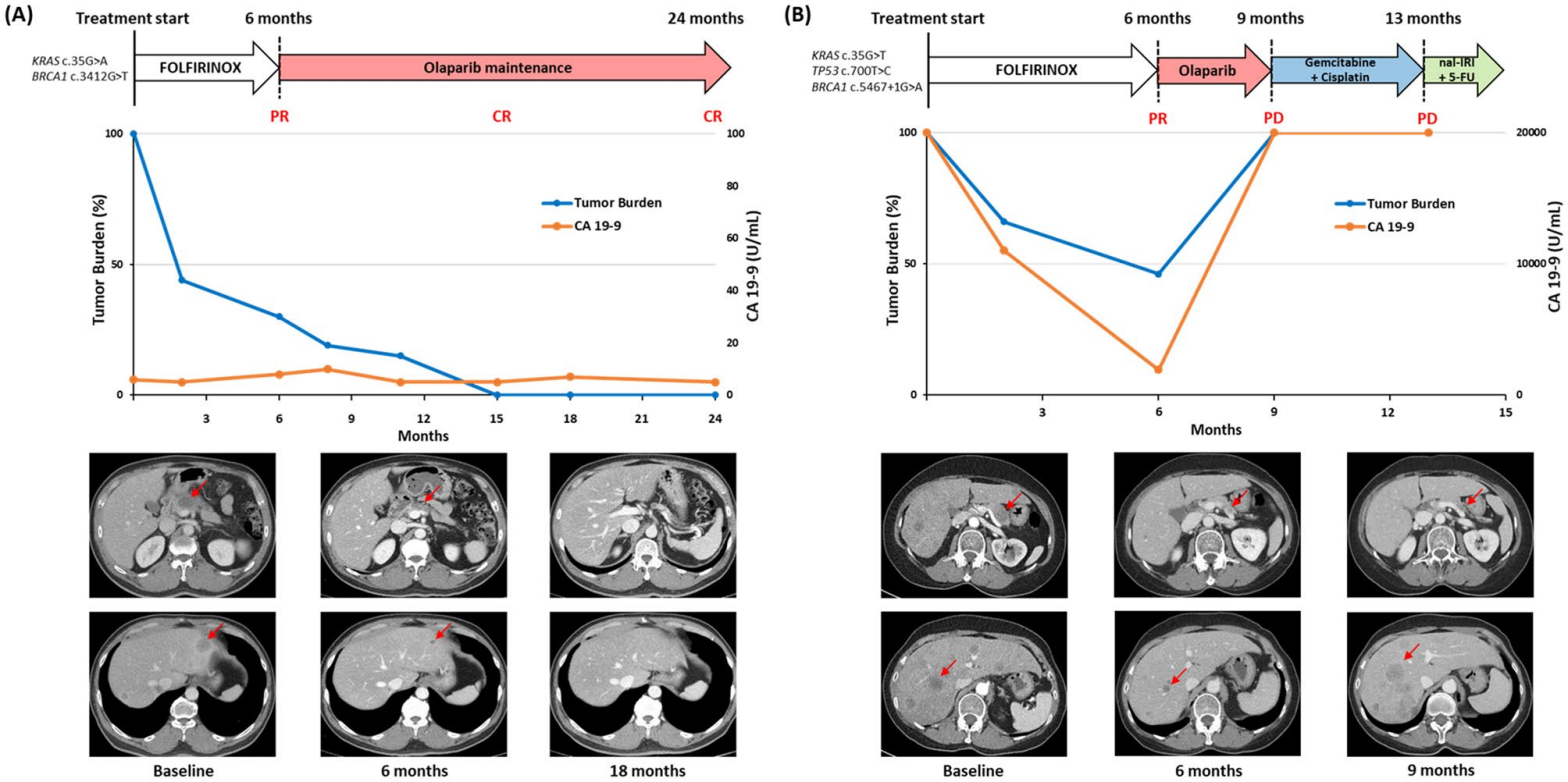
(**A**) and (**B**) Clinical course of two patients with germline pathogenic *BRCA1* mutations who were treated with PARP inhibitors. Changes in tumor burden (left y-axis) and the level of CA 19–9 (right y-axis) over time of treatment (months, x-axis) were presented together with specific computed tomography imaging.

The first patient who received olaparib maintenance treatment was a 54-year-old man with metastatic pancreatic cancer and a germline *BRCA1* mutation (c.3412 + 1G > T). He received nine cycles of FOLFIRINOX and displayed a partial response with a 70% reduction of the tumor burden versus baseline according to RECIST ver. 1.1 (Fig. 3A). Subsequently, he received maintenance treatment with a PARP inhibitor. The tumor burden gradually decreased during maintenance treatment, and the lesion became indiscernible on computed tomography scan after 1 year of PARP inhibitor treatment. He has consistently responded to PARP inhibitor therapy, which he has received for 18 months. He had a sister with ovarian cancer and a germline *BRCA1* mutation and a niece (the daughter of the sister with ovarian cancer) with breast cancer and a germline *BRCA1* mutation. He had three children, and genetic testing revealed a germline *BRCA1* mutation in one child.

The other patient was a 64-year-old woman with metastatic pancreatic cancer and a germline *BRCA1* mutation (c.5467 + 1G > A). She received nine cycles of FOLFIRINOX and displayed a partial response with a 50% reduction of the tumor burden versus baseline according to RECIST ver. 1.1 (Fig. 3B). She received maintenance therapy with a PARP inhibitor, but the tumor progressed after 3 months of treatment. Subsequently, considering the germline *BRCA1* pathogenic variant, she received third-line gemcitabine and cisplatin chemotherapy, but the tumor progressed after four cycles of treatment. She is currently receiving nanoliposomal irinotecan with fluorouracil as fourth-line therapy. She had a family history of variant cancers, including two first-degree relatives with gastric cancer (father and younger brother), two second-degree relatives with gastric cancer (father’s brother) and pancreatic cancer (mother’s brother), and a nephew (the child of a younger brother with gastric cancer) who was diagnosed with cholangiocarcinoma at the age of 28 years. Of her two children, one underwent germline *BRCA1* mutation testing, and the result was negative.

## Discussion

The present study reviewed the result of targeted gene profiling of patients with pancreatic cancer in a single tertiary center in Korea. Similar to the results of previous studies, which were mostly conducted in Western countries, *KRAS*, *TP53*, *CDKN2A*, and *SMAD4* mutations were most common, and the frequencies of these recurrent mutations were comparable to those in the TCGA dataset. In addition, potentially actionable mutations were found in more than 30% of patients, versus rates of 11–50% in previous studies^6–11^.

There have been several mutation profiling data of patients with pancreatic cancer in Asian populations^16–18^. Hong et al. conducted whole-exome and RNA sequencing in 83 patients with pancreatic cancer in Korea, and they attempted to demonstrate the molecular profiles and find potential prognostic biomarkers using molecular aberrations^16^. Moreover, Yang et al. and Zhang et al. also attempted revealing genetic characteristics of pancreatic cancer in two other studies in Chinese patients with pancreatic cancer using targeted NGS panels^17,18^. However, the number of studies on molecular profiling of pancreatic cancer in Asian populations is still much less than that in Western populations, and it is also difficult to integrate and compare these data with our study results because of significant differences in study objectives, clinical settings, and methodology. Therefore, further large-scale and standardized genomic studies on pancreatic cancer in Asian populations are necessary.

Mutational profiles in this study were analyzed by constructing a gene list for potentially actionable mutations in reference to the KYT study and classifying the mutations into two groups (highly actionable and modifies options)^9^. In the KYT study, 50% of patients had potentially actionable mutations, and 27% had highly actionable mutations. However, the rates of potentially and highly actionable mutations in our study were lower than those in the KYT study (31 and 14%, respectively). The gap between the two studies may be explained by multiple factors such as differences in the NGS panel (FoundationOne® panel [Cambridge, MA, USA] vs. customized NGS panel), the database used for variant interpretation, the number of patients with *KRAS* mutations (87% vs. 94%), and ethnicity. In addition, the percentage of actionable mutations varied in prior studies (11–50%), and a large targeted gene profiling study of more than 3500 patients with pancreatic cancer using the FoundationOne® panel also reported that 17% of patients had therapeutically relevant alterations, which was lower than the rate in the KYT study^6–11^.

Similar to previous studies of actionable mutations in pancreatic cancer^6,9–11^, mutations in DDR genes, such as *BRCA1*, *BRCA2*, and *ATM*, were the most frequent highly actionable mutations in this study. Several studies have demonstrated the survival benefit of platinum-based chemotherapy in such patients^12,19–21^. In addition, the recent POLO trial reported the clinical benefit of the PARP inhibitor olaparib as a maintenance treatment in patients with metastatic pancreatic cancer and germline *BRCA* mutations^15^. Two patients (2.3%) in this study were confirmed to have germline pathogenic *BRCA1* mutations. Both of them displayed partial responses to FOLFIRINOX, and one patient who harbored a stop-gain variant (c.3412G > T) exhibited a durable response to PARP inhibitor therapy for 18 months. Moreover, the number of patients with germline *BRCA* mutations who can benefit from PARP inhibitors, currently the only approved indication for targeted therapy in pancreatic cancer, might have been underestimated in this study because two other patients with *BRCA2* mutations (Tier I and Tier II, respectively) did not undergo germline testing. In addition, recent studies described the possibility of expanding the indication for treatments related to the DDR pathway, such as treatments targeting non-canonical DDR gene mutations, somatic DDR gene mutations, or genomic scars associated with DDR pathway deficiency through mutational signature or genomic instability analysis^19,22,23^.

Another potential targeted treatment in pancreatic cancer is immunotherapy. Based on the results of the KEYNOTE-158 study, the immune checkpoint inhibitor pembrolizumab is currently approved for treating any solid cancer with high MSI^24^. More recently, any solid cancer with high TMB, defined as > 10 mutations per megabase in the FoundationOne® CDx panel, was also approved as an indication for immune checkpoint inhibitor therapy^25^. In addition, *POLE* and/or *POLD1* mutations, which induced impairments in DNA proofreading and replication, were reported to be associated with the hypermutation phenotype and immune checkpoint inhibitor response^26–28^. As previously known that high MSI and pathogenic POLE and/or POLD1 mutations are rare in pancreatic cancer, they were not identified in our study^29^. High TMB was also rare in this study with only one patient (1.2%), but there remained pending issues such as variability of TMB results across different NGS panels or the proper cutoff level according to cancer types^30,31^. Therefore, further researches on TMB as predictive biomarker for immunotherapy in pancreatic cancer are necessary.

Our study had several limitations. First, this was a retrospective study, and selection bias was possible because the profiling test was not performed in all patients with pancreatic cancer. Second, the reliability of the SNUBH panel may be insufficient compared with other commercialized panels. However, the MSI status and *ERBB2* amplification, which were confirmed by other test methods in this study, revealed results that were consistent with the SNUBH panel (Supplementary Table S5). Third, only two patients underwent germline genetic testing following the result of tumor sequencing because germline pathogenic *BRCA1* and/or *BRCA2* mutations are the only approved indication for targeted therapy in pancreatic cancer. Fourth, potentially actionable mutations in this study were identified according to previous KYT study protocols; however, actionability can change over time. Therefore, the frequency of actionable mutations in this study may have been overestimated or underestimated in the present circumstances. Fifth, the number of patients who received targeted therapy or therapy based on a previously reported clinical trial according to the result of gene profiling was small. Finally, the sample size was insufficient to demonstrate the difference in the mutation profile according to various clinical characteristics. However, the present study had enough strengths as it attempted to compare the potentially actionable mutations with those of previous Western datasets using the same criteria as that used in Western datasets, and it further expanded the information in Asian populations.

In summary, targeted gene profiling in Korean patients with pancreatic cancer revealed similar frequencies of common recurrent mutations and potentially actionable mutations as recorded in Western data. Considering that approximately one-third of patients had at least one potentially actionable mutation and the number of actionable mutations can expand gradually, mutational profiling is expected to have significant clinical impact in patients with pancreatic cancer.

## Supplementary Information

Supplementary Information.

## Data Availability

The datasets generated during the current study are available from the corresponding author upon reasonable request.
